# Identification of Cell-Surface Molecular Interactions under Living Conditions by Using the Enzyme-Mediated Activation of Radical Sources (EMARS) Method

**DOI:** 10.3390/s121216037

**Published:** 2012-11-22

**Authors:** Koichi Honke, Norihiro Kotani

**Affiliations:** 1 Department of Biochemistry, Kochi University Medical School, Kohasu, Oko-cho, Nankoku, Kochi 783-8505, Japan; 2 Kochi System Glycobiology Center, Kochi University Medical School, Kohasu, Oko-cho, Nankoku, Kochi 783-8505, Japan; 3 Center for Innovative and Translational Medicine, Kochi University Medical School, Kohasu, Oko-cho, Nankoku, Kochi 783-8505, Japan; E-Mail: kotani@saitama-med.ac.jp; 4 Department of Biochemistry, Saitama Medical University, Iruma-gun, Saitama 350-0495, Japan

**Keywords:** molecular interaction, membrane microdomains, lipid rafts, EMARS, HRP, receptor tyrosine kinases, antibody array, proteomics

## Abstract

Important biological events associated with plasma membranes, such as signal transduction, cell adhesion, and protein trafficking, are mediated through the membrane microdomains. We have developed a novel method termed enzyme-mediated activation of radical sources (EMARS) to identify coclustering molecules on the cell surface under living conditions, which features a radical formation from an aryl azide reagent by horseradish peroxidase (HRP). For identification of molecules labeled by the EMARS reaction, antibody array system and mass spectrometry-based proteomics approaches are available. Spatio- temporally-regulated interaction between β1 integrin and ErbB4 involved in fibronectin-dependent cell migration and therapeutic antibody-stimulated interaction between FGFR3 and CD20 were discovered using the EMARS method.

## 1. Introduction

The life is maintained in harmony by continuous cross-talk between divergent elements everywhere in the organism. This cross-talking is performed at every rank of the hierarchy of life, such as inter-molecules, inter-cells, inter-tissues, inter-organs and inter-organisms, and elements at each rank form a complex network. According to the theory of complex dynamical systems, certain macroscopic phenomena emerge from interactions between microscopic elements via nonlinear, large-scale interactions, which is called self-organization. On this principle, a higher rank phenomenon is supposed to be generated. Hence, we believe that molecular interactions by self-organization give birth to creatures from chemical substances.

Molecular interactions on the cell surface are considered a suitable object for the study of self-organization in biology, because functional molecules are distributed non-randomly and exist as clusters in the nanometer-scale domains [1], which are dynamically formed and break up in a short time, and these processes are continuously repeated [2]. Furthermore, important biological events including signal transduction mediated by receptors, cell adhesion, and membrane protein trafficking take place in these membrane domains. These domains are called lipid rafts, caveolae, immunological synapses and so on in accordance with the biological situation. The sizes of the membrane domains assessed in a variety of living cells using fluorescence resonance energy transfer (FRET) and a single-molecule tracking analysis are 4 nm to mm-scale [1], 325–370 nm [3], 500–700 nm [4], and 600–800 nm [5]. Their sizes differ depending on the state of the cells and get larger when stimulated.

### Analytical Methods for Molecular Interactions on the Cell Surface

A variety of methods for cell surface molecular interactions have been developed. However, experimental evidence suggesting molecular interaction is insufficient until the molecular interaction is proven in living cells. Coimmunoprecipitation is a conventional technique to purify a static molecular complex. Detergent-insoluble membrane fractionation is routinely performed for preparation of lipid rafts that are enriched in cholesterol, sphingolipids and GPI-anchored proteins [6]. These biochemical methods are simple and easy, but experimental artifacts often happen during sample preparation. To guarantee the formation of molecular complex in living cells, cross-linking of the components with a chemical cross-linker under living conditions is required, yet the fixed length and shape of the cross-linkers tend to be a bottleneck associated with this method.

The morphological visualization with fluorescence microscopy or electron microscopy is reliable in that colocalization of distinct molecules is directly observed under physiological conditions. Especially, single molecule tracking technology allows us to visualize the real-time movement of molecular assemblies under living conditions [7]. However, testing molecules must be known before conducting such experiments. Therefore, these approaches are useless until coclustering molecules are identified.

## 2. Enzyme-Mediated Activation of Radical Sources (EMARS) System

### 2.1. Molecular Clustering Analysis Using the EMARS Method

**Scheme 1.**
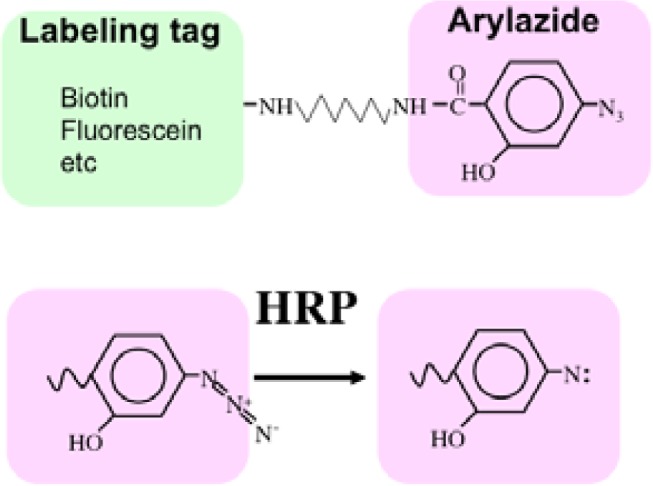
The enzyme-mediated activation of radical source (EMARS) reaction. The aryl azide group that is usually utilized for photoaffinity labeling is activated by horseradish peroxidase (HRP) to a nitrene radical. The aryl azide group can be conjugated with various labeling tags such as biotin and fluorescein.

Recently we developed a novel method termed enzyme-mediated activation of radical sources (EMARS) to identify coclustering molecules on the cell surface under living conditions [8–9], which features a radical formation from an aryl azide reagent by horseradish peroxidase (HRP) (Scheme 1). Radical formation was confirmed by an electron spin resonance (ESR) analysis, in which specific signal peaks of the adduct were observed using 5,5-dimethyl-1-pyrroline-N-oxide (DMPO) as a spin trap. These signals disappeared in the presence of a radical scavenger, ascorbic acid.

**Scheme 2.**
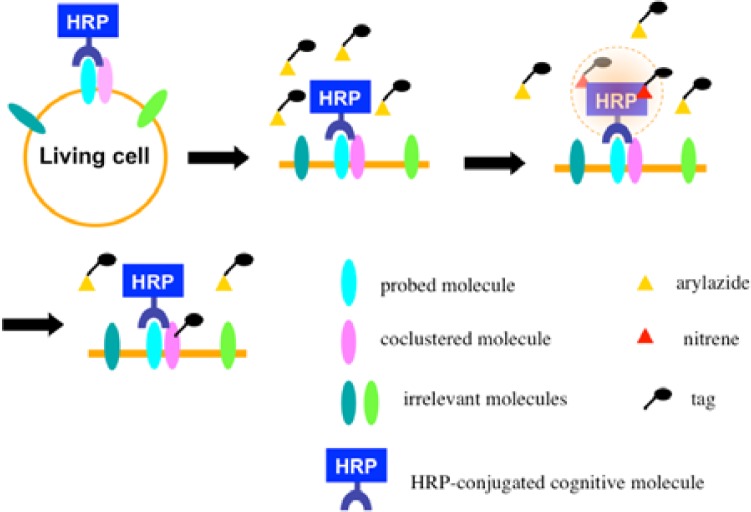
*In vivo* EMARS analysis.

Once the radical species are generated, the formed radicals are supposed to be short-lived and move within a limited area before decay. Therefore, we assumed that the reactive radicals generated by the EMARS reaction might attack the molecules located within a limited distance from HRP that was set on any molecule on the cell surface (probed molecule) to label the coclustered molecules with the probed molecule (Scheme 2). To address this hypothesis, the range of labeling around the probed molecule was assessed by means of immuno-electron microscopy. As the result, the molecules located within 200–300 nm around the probed molecule were labeled by the EMARS reaction [8]. Considering the range of labeling, the EMARS method may be suitable for identification of coclustering molecules in stimulated membrane domains.

First, an HRP-conjugated cognitive molecule such as an antibody is bound to the probed molecule on the cell surface of living cells. Next, an aryl azide reagent is added to the medium. The aryl azide group is activated to a nitrene radical by the HRP set on the probed molecule. The nitrene radical attacks certain reactive hydrogen sites or nucleophilic groups of adjacent molecules (coclustered molecules).

### 2.2. Identification of the Molecules Labeled by the EMARS Reaction

**Figure 1.**
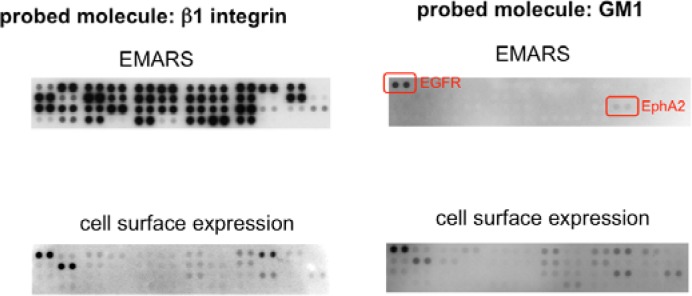
Coclustered molecules with β1-integrin and ganglioside GM1 revealed by the EMARS reaction. An antibody array analysis of the coclustered molecules of β1-integrin (**upper left panel***)* and ganglioside GM1 (**upper right panel**) in HeLa S3 cells. Antibodies against 42 kinds of receptor tyrosine kinases (RTKs) are spotted in duplicate on the array. Cell surface expression of RTKs was examined by labeling with *N*-hydroxysuccinimide biotin (**lower panels**). Adapted and used with permission from [8].

In order to identify the molecules labeled by the EMARS reaction, we first employed an antibody array system taking into account its high sensitivity and usefulness [8,10]. After the EMARS reaction in living cells, membrane proteins are solubilized and reacted with antibodies on an antibody array. Biotinylated molecules bound to the antibody array can be detected with streptavidin. For example, when anti-β1 integrin antibody was used as a probe of the EMARS reaction in HeLa S3 cells, many kinds of receptor tyrosine kinases (RTKs) were biotinylated (Figure 1, left panel) [8], suggesting that various RTKs cocluster with β1 integrin in living cells. Judging from the intensity of biotinylation on each RTK, probability of coclustering is different among RTKs, irrespective of their expression levels. In contrast to the case of β1 integrin, only two RTKs, EGFR and EphA2, were biotinylated when an HRP-conjugated cholera toxin B subunit (CTxB), which binds to ganglioside GM1, was used as a probe of the EMARS reaction (Figure 1, right panel) [8]. These results suggest that distinct molecular clusters can be distinguished by the EMARS method.

Originally, we developed the EMARS reaction using a commercially available biotin-tagged aryl azide as a labeling reagent [8]. It is all right as far as the labeled molecules are identified by the antibody array system for cell-surface membrane proteins such as RTKs. The antibody array system is very sensitive and easy to identify molecules, but only a limited number of antibodies are coated on the antibody array. To make the EMARS reaction a useful tool for a wide range of research concerning cell surface molecular interactions, labeled molecules should be identified by comprehensive methods such as mass spectrometry (MS)-based proteomics approach. To this end, usage of biotin-tagged aryl azide is of problem because this compound enters cells across the plasma membrane during the EMARS reaction. In the cell the biotin-tagged aryl azide is activated by endogenous enzyme(s) that have not been determined and subsequently bind to intracellular proteins. As a result, many nonspecific biotinylated proteins are detected on electrophoresis gels following the EMARS reaction, which makes it difficult to distinguish from the specifically labeled proteins by exogenous HRP [11].

**Figure 2.**
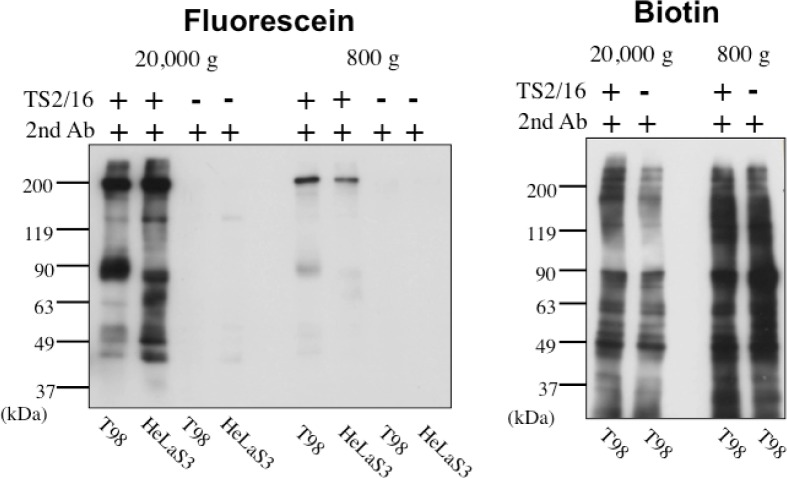
Suppression of the nonspecific labeling by endogeneous enzyme(s) in EMARS reaction with fluorescein-tagged aryl azide. EMARS reaction was performed with fluorescein-tagged aryl azide (**left panel**) or biotin-tagged aryl azide (**right panel**) after setting HRP on β1 integrin in T98 cells and HeLa S3 cells. After reaction, each sample was subjected to Western blotting with an anti-fluorescein antibody (**left panel**) or streptavidin (**right panel**). The 800 *g* fraction contains nuclei, mitochondria and peroxisomes. The 20,000 g fraction contains microsomes. The robust nonspecific labeling in the 800 g fraction in the sample with biotin-tagged aryl azide (**right panel**) disappeared in that with fluorescein-tagged aryl azide (**left panel**). Adapted from [11] and used with permission.

In order to solve this problem, we have tried to modify the labeling reagent and we found that fluorescein-tagged aryl azide, in which biotin tag is replaced with fluorescein, is activated by HRP but barely activated by endogenous enzyme(s) (Figure 2) [11]. This reagent has another advantage that the labeled molecules are directly detected with a fluorescence imager.

**Scheme 3.**
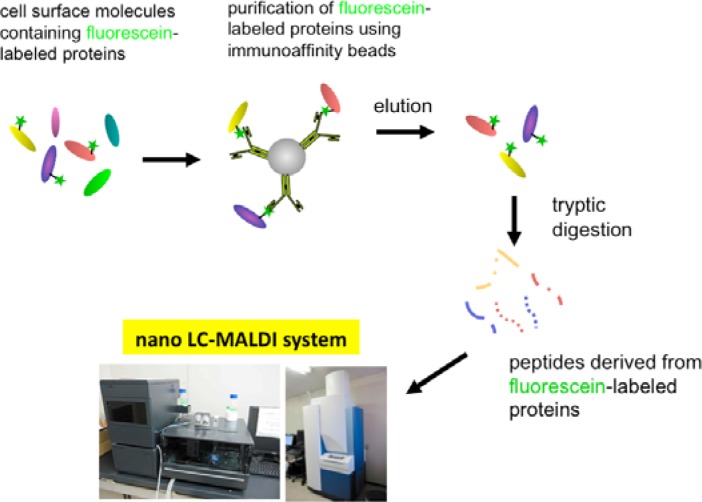
Identification of the EMARS product by proteomics technique. The fluorescein-labeled EMARS products are purified and concentrated by immunoaffinity chromatography with anti-fluorescein antibody-immobilized beads and identified by mass spectrometry (MS)-based proteomics analysis.

Eventually, we have established a method combined with the EMARS reaction using fluorescein-tagged aryl azide and MS-based proteomics analysis to identify cell-surface molecular interactions (Scheme 3) [11,12]. The fluorescein-tagged proteins resulting from the EMARS reaction were purified and concentrated by immunoaffinity chromatography with anti-fluorescein antibody-immobilized resins. The purified fluorescein-tagged proteins were subsequently subjected to an MS-based proteomics analysis.

## 3. Application of the EMARS Method for Identification of Cell-Surface Molecular Interactions

We present two examples of application of the EMARS method for identification of cell-surface molecular interactions. One is with regard to *cis*-interaction with a cell adhesion molecule that is induced by the stimulation of cell attachment, and the other is concerning molecular assembly around the antigen formed by binding with a therapeutic antibody.

### 3.1. Interaction with β1 Integrin and ErbB4 Induced by Cell Attachment

Cell adhesion and migration are basic biological events to form tissues and organs. These events are mediated by the contact between cell surface adhesion and extracellular matrix (ECM). Integrins are widely expressed cell-surface adhesion molecules that mediate cell attachment to ECM proteins, such as fibronectin, collagen and laminin. They also interact with molecules on their own membranes, and these *cis*-interactions play a crucial role in integrin-dependent cellular responses. We therefore analyzed what molecules interact with β1 integrin during biological events induced by cell attachment to different ECM proteins, by using the EMARS method [13].

**Figure 3.**
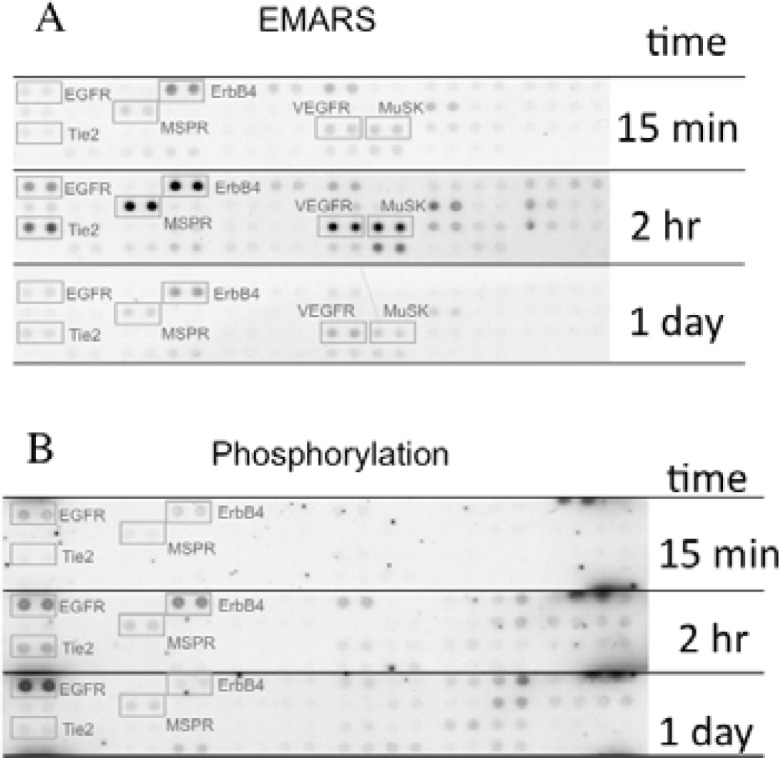
Interaction with β1 integrin and phosphorylation of RTKs. (**A**) Association between β1 integrin and RTKs in HeLa S3 cells. The EMARS reaction was performed at 15 min, 2 h and 1 day after seeding of HeLa S3 cells onto fibronectin using an HRP-conjugated anti-β1 integrin antibody as a probe and aryl azide-fluorescein as a labeling reagent. (**B**) Phosphorylation of RTKs. A part of the EMARS product was separately applied to antibody array and reacted with an anti-phosphotyrosine antibody. Adapted from [13] and used with permission.

We identified several candidates for integrin β1-associating partners that show apparent dependency on ECM species. For example, epidermal growth factor receptor (EGFR), ErbB4, macrophage-stimulating protein receptor (MSPR), Tie-2, vascular endothelial growth factor receptor 3 (VEGFR3), and muscle-specific kinase (MuSK) were detected in HeLa S3 cells seeded on fibronectin (Figure 3(A)). A time-course analysis of their clustering revealed that ErbB4, Tie-2, VEGFR3, and MuSK were predominantly detected at 2 h after seeding of cells. Moreover, tyrosine phosphorylation of ErbB4 reached a peak at 2 h after seeding the cells onto fibronectin and this timing coincided with that of the interaction with β1 integrin (Figure 3(B)). Accompanying with these findings, suppression of cell migration by a pharmacological inhibitor of the ErbB family receptors, PD168393 and an anti-ErbB4 neutralizing antibody, 12D8 was observed at 2 h after seeding. Taken together, it is deduced that interactions between β1 integrin and ErbB4 occur in a spatiotemporally-regulated manner, and such interaction contributes to the integrin-dependent cell migration.

### 3.2. Association of CD20 with FGFR3 Induced by Stimulation with Rituximab

Therapeutic antibodies are supposed to work mainly mediated by two immune reactions, namely the antibody-dependent cellular cytotoxicity (ADCC) and the complement-dependent cytotoxicity (CDC), but alternative mechanisms are also involved in their action. For instance, antibodies bind to their antigen receptors as a ligand and induce pharmacological effects such as cytotoxicity and cytokine secretion. These antibodies are called a signaling antibody and applied to clinical therapy. However, their action mechanisms remain to be elucidated in many signaling antibodies. Rituximab is reported to inhibit proliferation of lymphoma cells through an unknown CD20-mediated signal transduction pathway. Therefore, we investigated cell surface molecules involved in the CD20-mediated signal transduction pathway by using the EMARS method [14].

**Figure 4.**
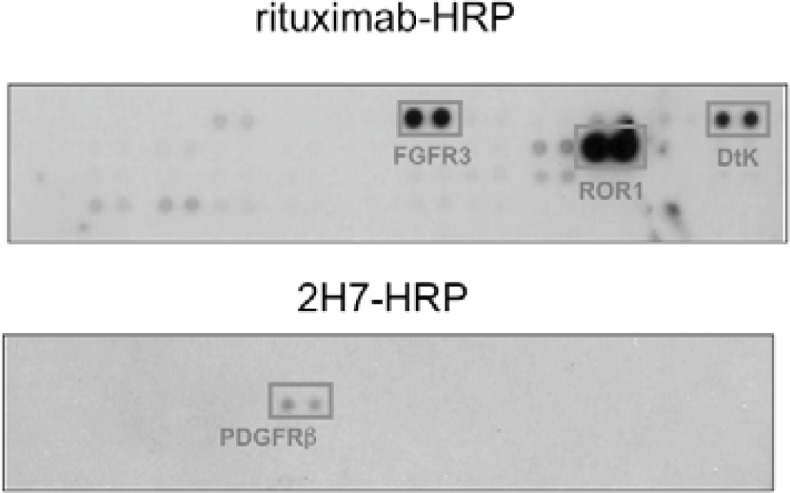
RTKs coclustered with rituximab-CD20 complex. The EMARS method was performed using HRP-conjugated rituximab or HRP-conjugated 2H7 antibody in Raji cells. The EMARS products from rituximab-HRP (*Rituximab-HRP*) and 2H7-HRP (*2H7-HRP*) treated Raji cells were applied to the RTKs antibody array. Adapted and used with permission from [14]. (This research was originally published in *The Journal of Biological Chemistry*. Kotani, N.; Ishiura, Y.; Yamashita, R.; Ohnishi, T.; Honke, K. FGFR3 associated with the CD20 antigen regulates the rituximab-induced proliferation inhibition in B-cell lymphoma cells. *J. Biol. Chem.* 2012, 287, 37109–37118. © the American Society for Biochemistry and Molecular Biology).

We found that under stimulation with rituximab, CD20 was associated with fibroblast growth factor receptor 3 (FGFR3) as well as some other receptor tyrosine kinases in Raji cells (Figure 4). On the other hand, under stimulation with a non-cytotoxic anti-CD20 antibody 2H7, CD20 was not associated with FGFR3 but with PDGFRβ. When the tyrosine kinase activity of FGFR3 was inhibited by a chemical inhibitor PD173074 or the expression of FGFR3 was knocked down by an siRNA, the proliferation inhibition by rituximab was attenuated. These results indicate that FGFR3 participates in the rituximab-dependent signal transduction pathway leading to proliferation inhibition.

## 4. Conclusions/Outlook

The EMARS system is a novel approach to identify cell-surface molecular clusters under living conditions. The advantages of this approach are as follows; (i) easy, high throughput, and no need for special equipment, (ii) applicable to systematic approaches such as proteomic analyses, (iii) applicable to studies not only on proteins, but also carbohydrate chains and membrane lipids. The EMARS reaction is therefore expected to become a powerful tool for a wide range of research concerning molecular interactions in membrane domains.

So far, we have performed the EMARS reaction using exogenously added HRP-conjugated antibodies and other cognitive molecules [8–14]. Since HRP-conjugated antibodies and tool kits for conjugation with HRP are commercially available, this system is simple and easy, but has some limitations. First, an excellent antibody toward the target molecule that works even after conjugation with HRP is needed. Second, binding of antibodies bring about artificial molecular clusters. Third, only cell surface molecular clusters can be examined as HRP-conjugated antibody cannot enter the cell. To improve the EMARS system for a wider range of research concerning molecular interaction, we are trying to establish a new generation of EMARS system, in which the EMARS reaction is catalyzed by HRP expressed in mammal cells by gene manipulation technology. We will publish about this new technology elsewhere some day. Obtaining comprehensive knowledge of the molecular interaction networks in membrane domains remains a challenge for the future.
